# Posttraumatic growth, posttraumatic stress and psychological adjustment in the aftermath of the 2011 Oslo bombing attack

**DOI:** 10.1186/1477-7525-11-160

**Published:** 2013-10-02

**Authors:** Ines Blix, Marianne Bang Hansen, Marianne Skogbrott Birkeland, Alexander Nissen, Trond Heir

**Affiliations:** 1 Norwegian Centre for Violence and Traumatic Stress Studies, NKVTS, Pb 181 Nydalen, 0409 Oslo, Norway

**Keywords:** Posttraumatic growth, Posttraumatic stress, PTSD, Adjustment, Life satisfaction

## Abstract

**Background:**

Experiencing potentially traumatic events is associated with psychological distress. However, some survivors also experience positive personal and psychological changes in the aftermath of trauma.

**Methods:**

The present study investigated perceived posttraumatic growth in 197 ministerial employees who were present at work during the 2011 Oslo bombing attack. The relationships between trauma-exposure, peritraumatic reactions and posttraumatic growth were studied. Moreover, the adaptive significance of posttraumatic growth was addressed.

**Results:**

The results showed that higher levels of trauma-exposure and immediate reactions were significantly related to perceived posttraumatic growth. No support for an adaptive significance of posttraumatic growth was found. On the contrary, posttraumatic growth was associated with higher symptom levels of posttraumatic stress. After adjusting for posttraumatic stress symptoms no association was found between perceived growth and work and social adjustment. However, perceived growth was associated with higher levels of life satisfaction.

**Conclusion:**

The present results are in line with previous findings indicating that perceived growth may be unrelated to psychological adjustment, and suggest that the concept and significance of posttraumatic growth should be interpreted with caution.

## Background

Some people experience positive personal and psychological changes in the aftermath of trauma, despite the psychological distress associated with potentially traumatic events. Post traumatic growth (PTG) has been defined as *“positive change that an individual experiences as a result of the struggle with a traumatic event”*[1]. Posttraumatic growth is thought to involve positive change in five major domains: greater appreciation of life and a change in sense of priorities, warmer and more intimate relationship with others, greater sense of personal strength, recognition of new possibilities or paths for one’s life, and spiritual development [2].

A number of empirical studies have demonstrated posttraumatic growth after several different types of traumatic events [3]. Among these, a handful of studies have shown PTG following terrorist attacks [4]. Posttraumatic growth has been found to be predicted by severity of exposure, both measured as objective characteristics of the traumatic event [5] and as self-report measures of immediate reactions [6]. Immediate reactions after trauma (e.g. helplessness, controllability and feeling of life threat) have been thought to be more important for growth than objective characteristics of trauma exposure [7,8].

Perceived growth is thought to arise as the result of a meaning making process in the aftermath of trauma. People try to make sense of what happened and cope with the emotional reactions and restore pre-trauma schemas. This is described as a process involving both automatic and intentional rumination, and the outcome is adaptation and growth [8]. Several theories have been put forward to explain why some individuals experience growth after trauma. Common for the theories is cognitive and emotional processing with the function of making sense of the traumatic event [9].

Posttraumatic growth entails positive change. The meaning making process thought to underlie posttraumatic growth, may provide new insights and new forms of meaning in life. In turn this process is believed to lead to higher levels of life satisfaction and psychological well-being. In line with this a recent study by Triplett and colleagues [10] showed that posttraumatic growth had a weak but significant direct association with life satisfaction.

However, the adaptive significance of PTG and its relationship to mental health and psychological adjustment in the aftermath of trauma is not clear and controversies exist on both measurement and conceptual issues. A review by Linely and Joseph [7] showed that findings on the relationship between PTG and mental health are inconsistent, and that positive, negative and null findings have been reported. In line with this, another review [3] pointed out that the literature was inconsistent with regards to the relationship between PTG and symptoms of posttraumatic stress (PTSD), and the relationship between PTG and symptoms of depression and other adjustment outcomes. Based on their review of the literature, Zoellner and Maercker [3] questioned whether PTG has an adaptive function. Furthermore, a meta-analysis by Helgeson, Reynolds and Tomich [11] showed that growth was associated with less depression and more psychological well-being measured as life satisfaction, self-esteem and positive affect. However, no association was found between growth and levels of anxiety, global distress, quality of life and physical health. Levels of PTSD symptoms were not addressed in this meta-analysis, but growth was associated with more intrusive and avoidant thoughts about the stressor. This review suggests that the relationship between growth and mental health is complex.

The relationship between PTG and mental health in survivors of terrorist attacks shows a similar complex pattern [4]. In a study of PTG after the 11^th^ September terrorist attacks, PTG was associated with higher levels of PTSD [12]. In line with this, two studies with Israelis who had experienced terrorist attacks showed that PTG was related to greater levels of PTSD symptoms [13], [14]. A recent longitudinal study following Israeli ex-prisoners of war and controls over 17 years, found that participants with PTSD had higher PTG levels across time than participants without PTSD [15].

Since PTG is measured by self-report questionnaires that require retrospective evaluation, it is difficult to know whether perceived positive changes after trauma reflect actual positive changes. It has been shown that perceived growth measured by the PTGI was not associated with actual growth in domains related to the questions in the PTG. While actual growth was associated with decreased distress, perceived growth was associated with increased distress [16]. These findings implicate that perceived growth and actual growth may be two different phenomena.

There is a need for studies that focus on whether perceived PTG corresponds with other measures related to posttraumatic growth. This leads up to the present study which aims to investigate the relationship between perceived growth and adaptive outcomes such as daily life functioning and life satisfaction.

### The present study

The aim of the present study was twofold. The first aim was to investigate the relationship between trauma-exposure and immediate reactions and PTG. We hypothesized that a higher degree of trauma-exposure and immediate reactions would be associated with higher levels of PTG. The second aim was to address the adaptive significance of PTG. More specifically, we investigated whether perceived growth was associated with less psychological problems, less impairment in daily functioning and higher levels of life satisfaction in the aftermath of trauma. Due to inconsistent findings in the literature, we made no specific predictions about the relationship between PTG, levels of PTSD symptoms, degree of impaired functioning and life satisfaction.

## Methods

### Participants

The present study investigated PTG in employees in the Norwegian Ministries in the aftermath of the terrorist attack in Oslo in July 2011. A politically oriented terrorist detonated a car bomb in the center of Oslo within the executive government quarter. Eight people were killed, at least 209 people were injured, and the bomb caused considerable damage to surrounding government buildings.

This study is a part of the study “Mental health and work environment factors in the aftermath of the Oslo terrorist attack July 22nd, 2011” [17]. Participants comprised employees in 14 of the 17 Norwegian ministries. 342 people were present at work in the ministries when the bomb exploded, all of them were asked to participate in the present study. Out of these, 207 (60.5%) participated in the study. Due to missing data on key variables 10 participants were not included in the present study. The total sample comprised 197 individuals (57.6%) (78 men, 119 females). The mean age was 44.8 (*sd* 11.7).

### Procedure

All participants were informed about the study by their respective ministries through internal meetings and emails and given the opportunity to withdraw. Data were collected online through a secure web-based questionnaire 9–10 months after the bomb explosion. All invited employees received a project specific identification number based on their social security number, and a personalized code to log on to the questionnaire. Only one administrative person who was not a member of the research team had the key to match a project ID number with the corresponding social security number, thus keeping the identity behind a response anonymous to the research team and the employer. The study was approved by the Regional Ethics Committee in Norway.

### Measures

#### Exposure

The participants were asked if they had witnessed people who were dead or dying, if they had witnessed people who were seriously injured, and if they were physically injured themselves. The answers to these three questions were added together giving a total exposure score ranging from 0–3, which are known to be major causal factors in the development of post-disaster stress [18].

#### Peritraumatic reactions

The participants were asked about their immediate reactions during the bomb explosion. Reflecting the DSM-IV A2 criteria, the participants were asked to what extent they experienced fear, helplessness and horror. The answers to these questions were added together to a total immediate reactions score ranging from 1–15. Each of the items was measured on a five-point scale: 1, not at all; 2, little; 3, moderate; 4, intense; 5 extreme [19].

### Posttraumatic growth inventory SF (PTGI-SF)

The PTGI-SF [20] is a 10-item questionnaire that assesses the degree of perceived positive change experienced after a traumatic event. Each question is answered on a 6-point Likert-scale, and the total score on the PTGI-SF ranges from 10 to 60. The PTGI-SF has shown good internal reliability [20]. In the present study Cronbach’s alpha was .89.

#### Posttraumatic check list-S (PCL-S)

The PCL-S is a 17-item self-administered questionnaire that assesses DSM-IV PTSD symptoms [21]. The participants are asked to indicate on a 5 point scale to which extent they had been bothered by the 17 symptoms over the course of the last month. A total symptom severity score (range = 17–85) is calculated by summing the scores from each of the 17 items. The items in the PCL-S were specifically linked to the bomb explosion for the purpose of the present study. In the present study Cronbach’s alpha was .95.

#### The work and social adjustment scale (WSAS)

The WSAS is a self-administered questionnaire used to measure the degree of impaired functioning [22]. The WSAS consists of five questions, scored from no impairment (0) to very severe impairment (8). A total score on impaired functioning, ranging from 0 to 40, is found by adding the scores on each of the 5 questions. The scale has been reported to be a reliable and valid measure of functioning [22]. In the present study Cronbach’s alpha was .97.

#### The cantril ladder of life

The Cantril Ladder of Life is a visual analogue scale [23] constructed to measure current life satisfaction. The ladder ranges from 0 to 10, where 0 reflects “the worst imaginable life” and 10 “the best imaginable life”. Participants were asked to indicate what step on the ladder that reflects their life at the moment.

#### Additional trauma-exposure

The participants were asked one question about whether or not they had witnessed or experienced other potentially traumatic experiences the last 12 months (for example natural disaster, serious accident, violence, robbery or assault).

### Statistical analyses

To investigate the relationship between trauma-exposure, peritraumatic reactions, levels of PTDS symptoms and PTG, bivariate and multivariate linear regression analyses were performed with PTG as the dependent variable. Age, gender, education, additional trauma-exposure, trauma-exposure during the bombing, and peritraumatic reactions were used as independent variables.

A similar approach was adopted to investigate the relationship between PTG, functioning in daily life, and life satisfaction. Bivariate and multivariate linear regression analyses was performed with work and social adjustment and life satisfaction as dependent variables and PTGI and PTSD symptoms as independent variables. The multivariate analyses were carried out when adjusting for age, gender and additional trauma-exposure. All tests were two-tailed and associations were considered significant if p < 0.05. Statistical analyses were conducted with SPSS version 20.0 for Windows (SPSS, Inc.).

## Results

Mean participants characteristics are presented in Table 1.

**Table 1:** Participants characteristics (n = 197)

**Variable**	**Mean**	**SD**	**Range**		**N (%)**
Exposure	1.22	.98	0	3	
- Witness dead or dying					67 (34)
- Witness injured					129 (65)
- Injured					50 (25)
Peritraumatic reactions	9.50	2.98	3	15	
- Fear	3.65	1.28	1	5	
- Helplessness	3.27	1.32	1	5	
- Horror	2.58	1.40	1	5	
Posttraumatic stress symptoms	33.95	15.04	17	85	
Life satisfaction	6.69	1.76	0	10	
Impaired Function	8.57	9.95	0	40	
Posttraumatic growth	28.92	9.58	10	60	

Table 2 shows that higher levels of trauma-exposure and higher levels of peritraumatic reactions were significantly associated with higher levels of PTG. This was also found after adjusting for gender and age. Women reported a significantly higher level of PTG.

**Table 2:** Stepwise linear regression predicting Posttraumatic Growth (n = 197)

	**Bivariate**	**Multivariate 1**	**Multivariate 2**
Exposure	.22**	.22**	.23**
Peritraumatic reactions	.22**	.22**	.16*
Additional exposure	.07		.05
Gender (women vs. men)	.19**		.16*
Age	-.10		-.04
R^2^		.10	.13
F		10.20***	6.87***

*p < .05, **p < .005, ***p < .001.

Posttraumatic Growth measured by PTGI-SF.

Bivariate regression showed that exposure, peritraumatic reactions, additional trauma-exposure and gender was positively associated with impaired work and social adjustment. When PTG and PTSD symptoms were entered into a multivariate analysis, only PTSD was significantly associated with impaired work and social adjustment. These associations remained significant after adjusting for age, gender and additional trauma-exposure (see Table 3).

**Table 3:** Stepwise linear regression predicting impaired work and social adjustment

	**Bivariate**	**Multivariate 1**	**Multivariate 2**
Posttraumatic growth	.21**	-.02	-.03
Posttraumatic stress	.87***	.88***	.84***
Additional exposure	.17*		.08*
Gender (women vs. men)	.36***		.11**
Age	-.02		.03
R^2^		.76	.88
F		302.26***	160.24***

*p < .05 **p < .005, ***p < .001.

Work and social adjustment measured by WSAS, posttraumatic stress by PCL, and posttraumatic growth by PTGI-SF.

Bivariate regression showed that level of PTSD symptoms was negatively associated with life satisfaction. No association between life satisfaction and PTG was found. When PTG and PTSD symptoms were entered into a multivariate analysis, the association between PTSD and life satisfaction remained significant, and higher levels of posttraumatic growth was significantly associated with life satisfaction. These associations remained significant after adjusting for age, gender and additional trauma-exposure (see Table 4).

**Table 4:** Stepwise linear regression predicting life satisfaction

	**Bivariate 1**	**Multivariate 1**	**Multivariate 2**
Posttraumatic growth	.06	.24***	.25***
Posttraumatic stress	-.55***	-.62***	-.61***
Additional exposure	.05		.01
Gender (women vs. men)			-.10
Age			.05
R^2^		.35	.36
F		52.19***	20.78***

***p < .001.

Life satisfaction measured by Cantril’s Ladder, posttraumatic stress by PCL, and posttraumatic growth by PTGI-SF.

## Discussion

Previous studies have demonstrated that perceived posttraumatic growth may occur after exposure to traumatic events. Few studies, however, have investigated to what extent posttraumatic growth is associated with adaptive functioning in daily life. We examined perceived growth in employees in the Norwegian Ministries after the Oslo bombing in 2011, and found that employees reported at least some degree of posttraumatic growth.

As expected, trauma- exposure and peritraumatic reactions were positively associated with perceived growth. This is in line with previous studies [7,8]. Perceived posttraumatic growth was related to higher levels of PTSD symptoms which also is in line with previous studies [12,13,15]. Based on previous studies showing a positive association between PTG and PTSD symptoms, Zoellner and Maercker [3] have questioned the adaptive function of PTG. Our findings give further support to this notion. Furthermore, the current findings showed that when adjusting for PTSD there was no significant relationship between PTG and functioning in work and social life. Hence, perceived growth does not seem to protect against impaired functioning in the aftermath of trauma.

Bivariate analyses showed no relationship between perceived growth and perceived life satisfaction. However, after adjusting for PTSD symptoms we found that higher levels of posttraumatic growth were associated with higher levels of life satisfaction. This is in line with a recent study that showed a weak but significant relationship between posttraumatic growth and life satisfaction [10].

In sum, the present study does not support the notion that PTG serves an adaptive function. It rather indicates that perceived growth is associated with impaired psychological health as indicated by higher PTSD scores. Moreover, PTG does not seem to buffer against impaired function in the aftermath of trauma. Even though PTG is not associated with how people are doing in terms of work and social adaptation, perceived growth is associated with higher life satisfaction given the same level of PTSD symptoms.

It may seem counter-intuitive that the people who report perceived growth are more likely to suffer from higher levels of PTSD symptoms. However, the relationship between PTSD symptoms and PTG might be explained by psychological mechanisms like cognitive dissonance. The harder trauma strikes the more one has to restore a sense of meaning and alleviate distress. A group of studies showed that trauma-exposed individuals reported greater personal change after a traumatic event compared to controls after a mild negative event. Interestingly, the perceived positive change was explained by that the trauma-exposed participants devaluated their pre-trauma attributes [24]. Hence, these findings suggest that the perception of posttraumatic growth is a result of a cognitive bias where trauma-exposed people tend to devaluate their past when making temporal comparisons, and this creates an illusion of positive change. As suggested by McFarland and Alvaro [24], this illusion might be a coping strategy that allow individuals to compensate for the negative effects of trauma.

Factors that might underlie both the feeling of perceived growth and higher levels of posttraumatic stress should be investigated in future studies. Rumination is one common factor thought to be important in the meaning making process after trauma and implicated in PTG [8] and in PTSD [25]. Another factor that should be explored in more depth is how the perceived centrality of a traumatic event can influence both perceived growth and symptoms of posttraumatic stress [26].

The present study has some limitations. Trauma exposure was operationalized as the number of episodes/events the participant experienced or witnessed during the bombing attack. Measuring degree of trauma-exposure is challenging, and a limitation of this method is that the same weight is attached to very different events, which can have different impact on different people. Furthermore, lifetime trauma-exposure was not measured in the present study. Due to the cross-sectional design of the present study, causality cannot be determined. It is well known that both levels of posttraumatic stress and posttraumatic growth may fluctuate with time. Hence, future longitudinal studies are needed to investigate paths to growth and PTSD, and to determine whether perceived growth has an adaptive significance.

In the present study, data was collected 9–10 months after the traumatic event, and hitherto we do not know how the relationship between growth and health outcomes would manifest over time. Time since trauma might be a relevant factor for the relationship between perceived growth and PTDS. For example, it has been proposed that in the immediate aftermath of trauma perceived posttraumatic growth can be seen as a cognitive coping mechanism, but after some time actual growth might develop [11].

Our sample of ministerial employees includes men and women in all adult age groups, and both posttraumatic stress and perceived growth showed considerable variation. Thus, we consider that the depicted relationships between the key variables contribute to knowledge about post disaster reactions in general.

The present study used a self-report questionnaire for measuring perceived growth after 22th of July. Answering questions about growth after trauma involves remembering how things were before the traumatic event and evaluating if things have changed since. As memory is constructive and particularly influenced by emotion [27], it can be questioned to what extent the answers reflect actual posttraumatic growth. Furthermore, the interplay between emotion and cognition connected to the traumatic event in the past can make it difficult to disentangle what is actual change after trauma, and what are cognitive biases.

## Conclusions

The phenomenon of post-traumatic growth is complex. Our findings question whether PTG serves an adaptive function, as we did not find data to support that increased levels PTG buffer against impaired functioning in the aftermath of a trauma. The need for more research, and in particular longitudinal research, is highlighted throughout the literature in this field. To determine the various trajectories of posttraumatic growth and to understand to what extent PTG involves various forms of cognitive processing, coping, and psychological adjustment, longitudinal research is indeed needed. Our findings highlight the importance of not interpreting posttraumatic growth as a solely positive or negative phenomenon. Future studies should explore the relationship between perceived growth and actual growth, and also the relationship between growth and increased vulnerability after trauma.

## Abbreviations

PTSD: Posttraumatic stress disorder; PTG: Posttraumatic growth; PTGI-SF: Posttraumatic growth inventory SF; PCL-S: Posttraumatic check list-S; WSAS: The work and social adjustment scale.

## Competing interests

The authors declare that they have no competing interests.

## Authors’ contributions

IB performed the literature review, carried out the statistical analyses and drafted the manuscript. MSB participated in the design of the study and helped to draft the manuscript. TH participated in the design of the study, the statistical analyses and participated in the write-up of the manuscript. MBH coordinated the data collection, participated in the design of the study and helped to draft the manuscript. AN coordinated the data collection and helped to draft the manuscript. All authors read and approved the final manuscript.
